# Digestion of Nucleic Acids Starts in the Stomach

**DOI:** 10.1038/srep11936

**Published:** 2015-07-14

**Authors:** Yu Liu, Yanfang Zhang, Ping Dong, Ran An, Changhu Xue, Yinlin Ge, Liangzhou Wei, Xingguo Liang

**Affiliations:** 1 College of food science and engineering, Ocean University of China, Qingdao 266003, China; 2 Department of Biochemistry and Molecular Biology, Qingdao University Medical College, Qingdao 266003, China

## Abstract

The ingestion of nucleic acids (NAs) as a nutritional supplement or in genetically modified food has attracted the attention of researchers in recent years. Discussions over the fate of NAs led us to study their digestion in the stomach. Interestingly, we found that NAs are digested efficiently by human gastric juice. By performing digests with commercial, recombinant and mutant pepsin, a protein-specific enzyme, we learned that the digestion of NAs could be attributed to pepsin rather than to the acidity of the stomach. Further study showed that pepsin cleaved NAs in a moderately site-specific manner to yield 3′-phosphorylated fragments and the active site to digest NAs is probably the same as that used to digest protein. Our results rectify the misunderstandings that the digestion of NAs in the gastric tract begins in the intestine and that pepsin can only digest protein, shedding new light on NA metabolism and pepsin enzymology.

It is well known that the nucleic acids (NAs) ingested from food are metabolized in the digestive tract by endonucleases, phosphodiesterases and nucleoside phosphorylase into oligonucleotides, nucleotides, and even free bases. Some of these metabolites can be absorbed by intestinal endothelial cells and are utilized for the salvage synthesis of NAs throughout the body, a process important for infant nutrition^1^ and for individuals with metabolic abnormalities^2^. Recently, it has been reported that ingested microRNA can regulate mouse gene expression *in vivo*^3^. This aroused our interest in the study of NA metabolism. The digestion of NAs is thought to start in the intestine and to be mediated by endonucleases^2^,^4^,^5^. Most textbooks and research papers describe a process in which histones are separated from DNA by pepsin and the acidity of the cellular environment, but few reports mention the role of the stomach in DNA metabolism. We noticed that much of our understanding of NA metabolism comes from studies conducted more than 30 years ago. However, it is well known that gastric juice is acidic, with a pH as low as 1.5 in some cases, and that depurination of DNA occurs to some extent under such acidic conditions. It is surprising that almost no discussion exists regarding NA breakdown in the stomach. This void prompted us to study in detail the metabolism of NAs in the stomach, which is also important for understanding the fate of NAs ingested from foods. Unexpectedly, we found that NAs could be digested by pepsin, which has been considered to be a protein-specific enzyme. This indicated that the digestion of NAs begins in the stomach rather than the intestine.

## Effect of gastric juice on DNA

**Figure 1:**
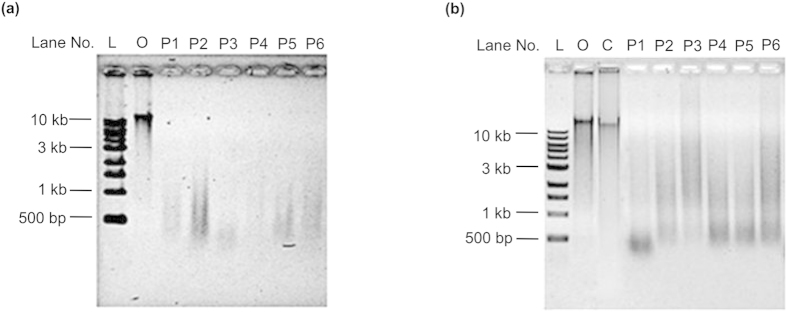
Digestion of salmon sperm DNA *in vitro* by human gastric juice. **a,** Original gastric juice from six individuals (Lanes P1-P6), of pH 1.52, 1.32, 3.57, 1.73, 1.51 and 2.28, respectively. **b**, Original gastric juices used in **a** were adjusted to pH 3.8 by the addition of NaH_2_PO_4_ buffer (pH 8.0). Lane L, DNA ladder; Lane O, original salmon sperm DNA; Lane C, a control of salmon sperm DNA treated by NaH_2_PO_4_ buffer (pH 3.8). Lanes P1-P6 contain salmon sperm DNA treated with six individuals’ gastric juice after pH adjustment. Digestion was carried out at 37 °C for 3 h and analysed on a 0.8% agarose gel.

We first examined the digestion of salmon sperm DNA by gastric juices from 6 individuals who had no serious gastric diseases. The pH of these gastric juice samples ranged from 1.32 to 3.57. As shown in Fig. 1a, much shorter fragments (<1 kb) of DNA were observed after treatment with the juices for 3 h, demonstrating that DNA could be destroyed by gastric juice. Considering that depurination occurs in strongly acidic conditions (pH < 3) and that breaking phosphodiester bonds becomes easier at abasic sites^6^, the digestion of NAs was further studied after adjustment of the pH of the gastric juices to 3.8 in order to investigate whether the digestion observed could be attributed to acidity (Fig. 1b). Interestingly, the digestion of DNA occurred again for all six samples, while no degradation of DNA was observed for the control in acidic buffer (pH 3.8). At this pH, less than 0.2% depurination occurred in 3 h^7^, indicating that the depurination did not contribute to the DNA digestion at pH 3.8. It can be concluded that the DNA digestion is not due to the acidity of pH 3.8. We also examined the effect of acidity on destroying DNA and found that acidic buffers with a pH more acidic than 2.5 could not break NAs under the same conditions (Supplementary Fig. 1b and 2b). When porcine pepsin was added to the above acidic buffers to simulate gastric juice, much shorter DNA bands were observed, indicating that DNA might be digested by an enzymatic component of gastric juice (Supplementary Fig. 1a and 2a). Porcine pepsin was used here because the alimentary tracts of pigs are genetically similar to those of *Homo sapiens*. Although breakage of NAs was observed after 24 h in the absence of pepsin when pH < 2.5 (Lane 2 in Supplementary Fig. 1b), much more digestion occurred when pepsin was present (Lane 1 and 2 in Supplementary Fig. 1a, Lane 1 in Supplementary Fig. 2a). This result was further confirmed by HPLC analysis (data not shown). The weak bands obtained at lower pHs (<2.5) might be due to difficult staining with dyes at abasic sites caused by depurination (Supplementary Fig. 1a, Lane 1). Few enzymes show activity in the gastric juice, because most proteins are denatured under strong acidic conditions and are destroyed by pepsin. Furthermore, it has been reported that the pH of gastric juice becomes higher than 3.0 after food intake^8^. Accordingly, it can be deduced that pepsin itself in the stomach might digest DNA or accelerate its digestion over a wide range of pHs.

## Effect of pepsin on nucleic acids

**Figure 2:**
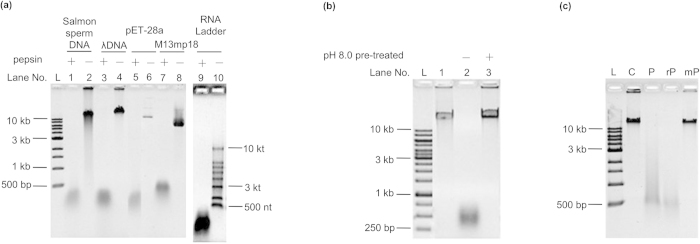
Validation of nucleic acid digestion by pepsin. **a**, Digestion of various DNA and RNA by commercial porcine pepsin. Lane 1, 2: salmon sperm DNA; Lane 3, 4: λ DNA; Lane 5, 6: pET-28a; Lane 7, 8: M13mp18; Lane 9, 10: RNA ladder. Other conditions: 4.0 mg ml^−1^ of pepsin, NaH_2_PO_4_ buffer (25 mM, pH 3.8, including 200 mM NaCl), 37 °C, 5 h. For RNA, the digestion time was 1 h. **b**, Effect of alkaline conditions on pepsin NA digestion. Lane 1, original λ DNA; Lane 2, digested by active pepsin; Lane 3, digested by NaOH-pretreated pepsin. **c,** Digestion of λ DNA by commercial porcine pepsin (Lane P), recombinant pepsin (Lane rP) and mutant pepsin (Lane mP). Conditions: 0.15 mg ml^−1^ enzymes, pH 3.8, 37 °C, 12 h. A 0.8% agarose gel was used for electrophoresis.

Pepsin is a proteinase that hydrolyses the amide bonds within proteins, and its ability to digest NA is novel and unusual. To better understand this unexpected ability, the breakdown of NAs by pepsin was studied in detail. At first, the digestion of various DNA and RNA sequences by pepsin was investigated. As shown in Fig. 2a, digestion by pepsin was observed for DNA extracted from salmon sperm, bacteriophage λ, plasmid pET-28a, and M13mp18 phage. The pH of these reactions was maintained at 3.8 in buffer solution containing 25 mM NaH_2_PO_4_ and 200 mM NaCl^9^,^10^. After digestion at 37 °C for 5 h, fragments shorter than 1 kb were observed (Lane 1, 3, 5, 7 in Fig. 2a). Interestingly, efficient digestion of RNA by pepsin was also observed (Lane 9 in Fig. 2a).

It has been reported that pepsin loses its activity irreversibly after treatment at pHs above 8.0^11^. Considering that nuclease contamination may cause the observed digestion, we examined whether digestion could occur after pepsin inactivation at pH 8.0. Interestingly, after the pepsin solution was adjusted to pH 8.0 and maintained for 30 min, evidence of λ DNA digestion at pH 3.8 was completely lost (Lane 3 in Fig. 2b). Similar results were also obtained for salmon sperm DNA as the substrate (Supplementary Fig. 3a) and when gastric juice was used to digest salmon sperm DNA (Supplementary Fig. 3b). Most nucleases do not lose activity at such a neutral pH (Supplementary Fig. 4 and 5), indicating that the digestion of DNA was caused by pepsin itself.

Commercial porcine pepsin was extracted from porcine gastric mucosa (Supplementary Fig. 6a and b). Nuclease contamination was difficult to remove during the extraction process, so we continued our experiments with recombinant pepsin. Expression of the cloned pepsin gene was carried out in *Pichia Pastoris* X-33 yeast cells with the **pPICZα A** vector. For comparison, a pepsin mutant was also cloned and expressed with two aspartic acids residues in the active site changed to alanine^12^. Purity of the recombinant pepsin (rP) and the mutant pepsin (mP) was above 98% (Supplementary Fig. 6c, 6d and 6e). As shown in Fig. 2c, the recombinant pepsin (Lane rP) had similar digestion activity to the commercial porcine pepsin (Lane P), but the mutant pepsin (Lane mP) did not show any activity. We also checked the ability of all pepsins to digest hemoglobin^13^, and rP showed similar activity as commercial porcine pepsin but mP did not. This suggested that even after recombinant expression and purification process some contamination existed but that these impurities had no effect on NA digestion. These results indicated that the proteinase active site in pepsin also exhibited the ability to digest NAs, although we could not exclude completely the possibility that some impurities in commercial pepsin supply nuclease activity.

## Mechanism for pepsin digestion of nucleic acids

**Figure 3:**
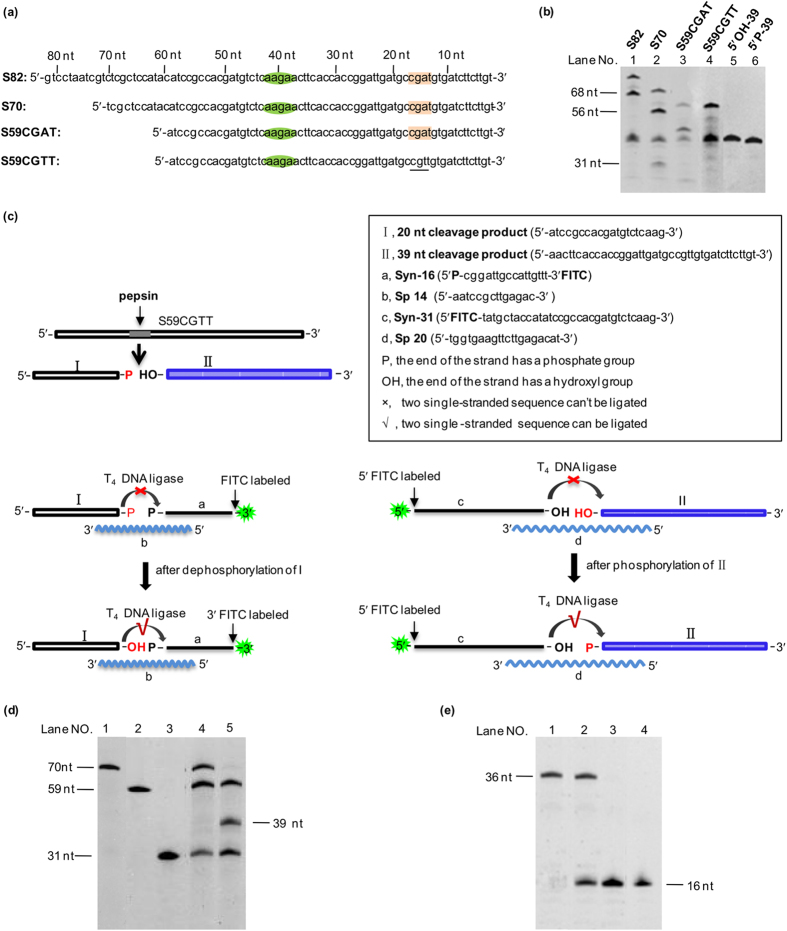
Analysis of pepsin cleavage sites and structure of cleaved fragments. **a,** Sequences of ssDNA. **b,** Digestion of ssDNA (1 μM) with specific sequences by pepsin. Lane 1, S82; Lane 2, S70; Lane 3, S59CGAT; Lane 4, S59CGTT; Lane 5, synthetic 5′OH-39 nt; Lane 6, synthetic 5′P-39 nt. **c,** Determination of 3′ or 5′ phosphorylation of the digestion products. **d,** Characterization of the 5′ end of the S59CGTT (3′ FITC) digestion product. Lane 1, Synthetic 5′ FITC-70; Lane 2, Synthetic S59CGTT (3′ FITC); Lane 3, Syn-31; Lane 4, the phosphorylated 39 nt cleavage product ligated with Syn-31. Lane 5, the 39 nt cleavage product without phosphorylation ligated with Syn-31. The malor ratio of Syn-31: Sp20: S59CGTT (3′ FITC) was 2 : 2 : 1. **e,** Characterization of the 3′ end of the S59CGTT digestion product. Lane 1, synthetic 5′ FITC-36 nt DNA. Lane 2, ligation of the native digestion product. Lane 3, ligation of the 3′ end dephosphorylated product. Lane 4, Syn-16. The malor ratio of Syn-16: Sp14: S59CGTT was 2 : 2 : 1. Electrophoresis was performed on a 20% denaturing acrylamide gel.

The phosphodiester bond in DNA is quite different from the amide bonds of proteins. By what mechanism does pepsin, a protease, act on NAs? Here, we further investigated the characteristics of this interesting phenomenon in detail. At first, we noticed that the length of digestion products were usually above 100 bp (or nt). Even when the reaction time was extended to 24 h, almost no oligonucleotide shorter than 100 bp (or nt) was observed by both agarose electrophoresis (Supplementary Fig. 7) and PAGE electrophoresis (data not shown). Therefore, pepsin may digest NAs in a sequence-specific manner rather than in a random pattern. As DNA denatures to a single-stranded state under acidic conditions, we employed several ssDNA (single-stranded DNA) sequences, and the cleavage with moderate sequence specificity was obtained (data not shown). Among these ssDNA, we selected S82, an 82 nt long sequence from pUC-18 plasmid, to determine the cleavage sites by pepsin, and found that S82 was cleaved mainly at two specific sites (Fig. 3b). Based on the S82, new segments were designed, which were 70 nt long and 59 nt long and had the same 3′ sequence as that of S82. For S59CGAT and S59CGTT, only one base was different at the 15^th^ position from 3′-end (Fig. 3a). Interestingly, digestion by pepsin showed that a clear band corresponding to a 39 nt long fragment was observed from the 59 and 70 nt long ssDNA substrates (Fig. 3b), indicating that the 39^th^ phosphodiester bond from the 3′ end was cleaved. For S82, a smear band at approximately 40 nt was observed (Lane 1, Fig. 3b). Because the 3′ end of S82 has the same sequence as other ssDNAs, the 39 nt long fragment at the 3′ end should be the same, and the left fragment at the 5′ end should be 43 nt long. The presence of these 43 nt and 39 nt long fragments may cause the smear. For S70, a weak band was observed at approximately 31 nt, and could be attributed to the fragment at the 5′ end (Lane 2, Fig. 3b). Thus, the cleavage site was determined to be 5′-AAG↓AA-3′ (Fig. 3a). For S59CGAT, a weak band at approximately 45 nt was also observed, but the band disappeared when S59CGTT was used. As only the base at 15^th^ position from 3′ end was changed from A to T, it could be deduced that the cleavage occurred at CGA↓T for S59CGAT. Therefore, cleavage should also occur for S70 and S82, and the resulting products should be 56 nt and 14 nt long for S70, and 68 nt and 14 nt long for S82. As shown in Fig. 3b, a band at approximately 56 nt was found for S70, while a band at approximately 68 nt was found for S82. These experiments confirmed that the assignment of cleavage to the 14^th^ position from the 3′ end (CGA↓T) was correct. Meanwhile, a 25 nt long band in S82, S70 and S59 should appear following double cuts between the 5′-AAG↓AA-3′ and CGA↓T sites^14^. However, it was invisible in the gel, probably due to the difficult staining. We excluded the possibility that the sequence between the cleavage site “AAGAA” and “CGAT” was also cleaved, because there was no band between the two bands of 68 nt and ∼40 nt long in the case for cleavage of S82.

The next question is whether the phosphate is attached to the 3′ end or 5′ end of the obtained fragments. To address this, a series of experiments were designed^15^ (Fig. 3c). As shown in Fig. 3c, if a fragment has a 3′-phosphate, its 3′ end will not be ligated to another synthesized oligo-DNA by T4 DNA ligase, which can only catalyse the ligation of 5′-phosphate and 3′-OH. It follows that the other fragment will have 5′-OH and can be ligated after phosphorylation. If a fragment has a 5′-phosphate, it can be ligated directly to another synthesized oligo-DNA via its 3′-OH by T4 DNA ligase. The ligation results were shown in Fig. 3d,e. As shown in Fig. 3d (Lane 5), the 39 nt cleaved fragment could not be ligated to Syn-31 (Fig. 3c) without phosphorylation. In the case of phosphorylation, as expected, the band for the 39 nt cleaved fragment disappeared (Fig. 3d, Lane 4), indicating that the cleavage fragment had free 5′-OH without phosphate. The Syn-31 was not completely ligated because the molar ratio of Syn-31: Sp20: S59CGTT (3′ FITC) was 2 : 2 : 1 in the ligation experiment, and at most 50% of Syn-31 could be ligated to the cleaved fragment by pepsin (Fig. 3d, Lane 4). However, after dephosphorylation, the 20 nt long cleavage product could be ligated to a synthetic 16 nt long (Syn-16) oligonucleotide with a 5′-PO_4_ on a splint DNA (Sp14) (Lane 2, Fig. 3e), but no ligation was observed without dephosphorylation treatment (Lane 3, Fig. 3e). Accordingly, the 20 nt long fragment had a 3′-phosphate. Thus, it could be concluded that pepsin digests DNA to give one fragment with a 3′-phosphate and another fragment with a 5′-OH.

**Figure 4:**
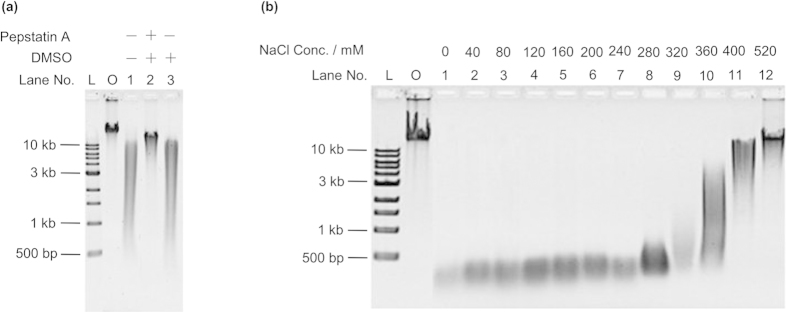
Inhibition of pepsin-induced DNA digestion by pepstatin A and salts. **a**, Effect of pepstatin A on digestion of λ DNA at pH 3.8 for 5 h. Lane L, DNA ladder; Lane O, original λ DNA; Lane 1, no pepstatin A; Lane 2, 250 μg ml^−1^ pepstatin A and 2.5% DMSO; Lane 3, only 2.5% DMSO. Pepstatin A was dissolved in DMSO. **b**, Effects of various concentrations of NaCl on NA digestion at pH 3.8 for 5 h. A 0.8% agarose gel was used for electrophoresis.

Next, we examined whether the cleavage of DNA by pepsin occurred at the same active site used to digest protein. Pepstatin A is an inhibitor of pepsin^16^, and we assayed its effect on the digestion of DNA by pepsin. Interestingly, when pepstatin A was added, the digestion of λ DNA was efficiently inhibited (Lane 2, Fig. 4a). The DMSO used to dissolve pepstatin A had no inhibiting effect on DNA digestion, indicating that the pepstatin A itself inhibited the digestion (Lane 3, Fig. 4a). Similar results were obtained when salmon sperm DNA was used as the substrate. We also found that pepstatin A could inhibit DNA digestion by gastric juice (data not shown). Thus, the above results indicated that pepsin digests DNA at the same active site used for digesting proteins.

It has been reported that the activity of pepsin for digesting proteins can be blocked by high concentrations of NaCl^17^. As shown in Fig. 4b, when NaCl was higher than 320 mM, digestion of λ DNA was greatly slowed (Lane 9), and almost no digestion was observed when NaCl concentration was increased to 520 mM (Lane 12). We observed the same inhibitory effect of NaCl on the digestion of the plasmid pET-28a (Supplementary Fig. 8). A similar inhibitory effect of NaCl on both on protein^15^ and NA supports the conclusion that the active site for their digestion is probably the same.

**Figure 5:**
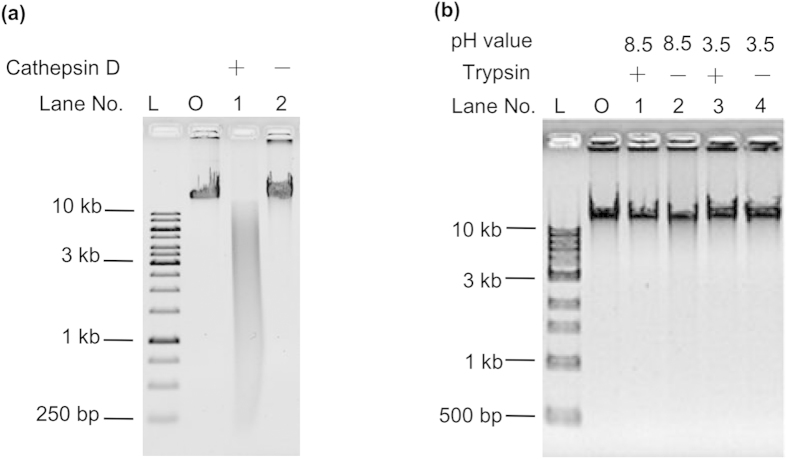
Digestion of λ DNA by other proteinases. **a**, Cathepsin D. **b**, Trypsin. Lane O, original λ DNA; Lane L, DNA ladder. A 0.8% agarose gel was used for electrophoresis.

Finally, Cathepsin D^18^, another aspartic proteinase, was used to digest DNA. Interestingly, Cathepsin D could also digest DNA to some extent (Lane 1, Fig. 5a). On the other hand, when a trypsin^19^ was used, which is a protease belonging to the serine protease family, no cleavage was observed at its optimum pH (pH 8.5) or an acidic pH (pH 3.5) (Fig. 5b). These results indicated that DNA might be recognized and cleaved by the active site of most aspartic proteinases, but not by other kinds of proteinase.

## Discussion

All the evidence presented in this study shows that NAs can be digested by pepsin. It is possible that some NA digestion could be attributed to nucleases contaminating pepsin or gastric juice. However, even if nucleases were present, their efficiency would be very low due to the acidic conditions and the digestion by pepsin. Even if some nucleases, such as DNase II, could survive under acidic conditions, they did not lose their activity at natural pH (pH 7.0) (Supplementary Fig. 5).

Our finding that pepsin can digest both protein and NAs is unprecedented. As noted by Sigma-Aldrich (which supplies the pepsin used in this study): “Pepsin hydrolyzes only peptide bonds, but does not hydrolyze non-peptide amide or ester linkages”, indicating that pepsin was thought to work on proteins only, although porcine pepsin had been recently reported to hydrolyse glycosidic bonds^20^. The possible reasons for NA digestion by pepsin are discussed in detail below.

Pepsin belongs to the aspartic class of proteinases, characterized by their ability to hydrolyse peptide bonds where an aromatic amino acid residue such as phenylalanine, tyrosine or tryptophan is present. Nucleobases are similar in size and molecular structure to these three aromatic residues, and the DNA duplex dissociates at least partly to a single-stranded state under acidic conditions below pH 4.0. Although the phosphodiester bond is quite different from the peptide bond, ssDNA has a structure similar to that of peptide chains containing aromatic residues. Thus, pepsin may recognize and bind to nucleobases and hydrolyse the phosphodiester bond at its active site. On the other hand, there are few enzymes that can catalyse two different reactions efficiently. To assess the efficiency of hydrolysis by pepsin with NA as the substrate, we measured kinetic parameters at pH 3.0 using the S82 sequence as the substrate and found the *K*_m_ and *k*_cat_/*K*_m_ values to be 55.6 ± 2.2 μM (standard deviation) and (1.05 ± 0.1) × 10^−2^ s^−1^ mM^−1^, respectively (Supplementary Fig. 11). However, at the similar pH, the *K*_m_ and *k*_cat_/*K*_m_ values for protein were 40 ± 1 μM and 2640 ± 40 s^−1^ mM^−1^ 21. It is apparent that the catalysis efficiency of pepsin on NA is approximately 10,000 times lower than that on protein. This is consistent with our results indicating that a relatively high concentration of pepsin was required for the efficient hydrolysis of NAs, and the efficiency was much lower than those of standard nucleases. The mechanism of NA digestion by pepsin requires more detailed study, and its clarification may contribute greatly to enzymology.

NAs can be digested by pepsin, which is the major enzyme found in gastric juice. Therefore, the stomach may play an important role during NA digestion, upending the textbook dogma that NA digestion begins in the intestine. Our results showed that the conditions in stomach may be sufficient for digesting NAs. Pepsin is the primary enzyme in the stomach, and its concentration in stomach falls in the range of 0.5–1.0 mg ml^−1^ 22,23. We found that the digestion of NAs occurred even when the pepsin concentration was as low as 1.0 μg ml^−1^ (Lane 8, Supplementary Fig. 9). We also found that the presence of hemoglobin did not inhibit NA digestion (Supplementary Fig. 10). It can be deduced that NAs can be—and may tend to be—digested in the stomach even after ingestion with other protein. Furthermore, the concentration of NaCl in food is usually lower than 200 mM, and this concentration does not greatly affect the digestion of NAs by pepsin in stomach (Fig. 4b, Supplementary Fig. 8).

**Figure 6:**
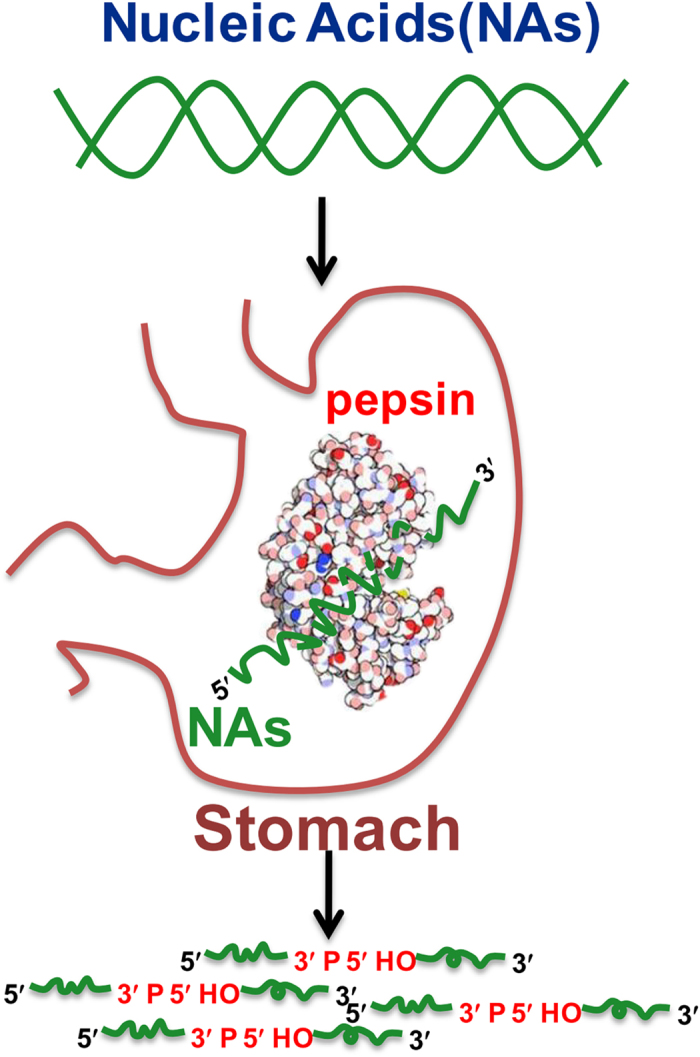
Pepsin in the stomach plays a key role in digesting NAs. We found that human gastric juice was able to significantly digest ingested NAs *in vitro*. Further study demonstrated that the dominant enzyme in gastric juice, pepsin, was responsible for this activity. Pepsin digested NAs at specific sequences, and the digested fragments had a 3′-phosphate and 5′-OH. The active site for cleaving phosphodiester bonds was the same as that used for digesting peptide bonds. Our findings correct the misunderstanding that NAs are not digested before reaching the small intestine. We propose that NAs are digested in the stomach rather than the intestine.

In conclusion, the dominant enzyme in gastric juice, pepsin, plays a key role in the digestion of NAs. Pepsin can digest NAs of specific sequences, and the digested fragments have 3′-phosphate and 5′-OH ends. The active site for cleaving phosphodiester bonds seems to be the same as that used to digest peptide bonds. Our study suggested that the digestion of NAs starts in the stomach rather than the intestine (Fig. 6). In addition, the breakdown of NAs in the stomach may accelerate their digestion by nucleases in the intestine. Efficient NAs digestion may help us to utilize the ingested NAs as well as protect us from genetic invasion. Our results are important for understanding the effects of genetically modified food on our health.

## Methods

### Preparation and storage of reagents and solvents

Gastric juices were gifts from the Department of Gastroenterology at the Affiliated Hospital of the Medical College of Qingdao University and the Qingdao Municipal Hospital. Original gastric juices were transferred and stored at −20 °C after they left the stomach.

Pepsin was purchased from Sigma-Aldrich (P6887) and was dissolved in water to a concentration of 40 mg ml^−1^. Digestion buffers for pepsin were prepared as stock solutions of 800 mM NaCl and 100 mM NaH_2_PO_4_ in H_2_O, and the pH was adjusted to the required values by the addition of 1 M HCl or 1 M NaOH.

Salmon sperm DNA (>10 kb) was purchased from Sigma-Aldrich Co., Ltd (31149) and dissolved in H_2_O to 300 μg ml^−1^. λ DNA (48.5 kb) was purchased from Thermo Fisher Scientific (Fermentas, SD0011) with 300 μg ml^−1^. Storage buffer for λ DNA supplied together with DNA was 10 mM Tris-HCl (pH 7.6) containing 1 mM EDTA. Plasmid pET-28a (5.4 kb) was purchased from EMD Chemicals Inc. (Novagen, D00131614) with concentration of 500 μg ml^−1^, and was diluted into 210 μg ml^−1^ before use. M13mp18 DNA (7.2 knt, ssDNA) was purchased from New England BioLabs (N4040S) with 250 μg ml^−1^. Stock buffer for M13mp18 DNA supplied together with DNA was 10 mM Tris-HCl (pH 7.5) containing 1 mM EDTA, and stock concentration for M13mp18 DNA was 90 μg ml^−1^. RNA ladder (0.5–10 knt) with 500 μg ml^−1^ was purchased from TaKaRa Biotechnology (Dalian) Co., Ltd. (D523A). It was diluted into 42 μg ml^−1^ by H_2_O before use. Their sequences were listed in Fig. 3 and Supplementary Fig. 8b. They were diluted into 42 μg ml^−1^ by H_2_O before use. Single- stranded DNA (ssDNA), such as S82, S70, etc., were supplied by Shanghai Sangon Biological Engineering Technology & Services. They were dissolved in H_2_O to a concentration of 10 μM, and the final reaction concentration was 1 μM.

Fresh porcine stomach was obtained from a local meat packing company and immediately cooled on ice. The stomach mucosa was removed and kept at –80 °C until use. The plasmid vector **pPICZα A** was purchased from Invitrogen (San Diego, CA). The *Pichia Pastoris* X-33 strain was purchased from CICC (China Center of Industrial Culture Collection, Beijing, China). *E. coli* TOP10, Trizol, TIANscript RT Kit and TIANpure Mini Plasmid Kit were purchased from TIANGEN Biotech Co., Ltd (Beijing, China). Polymerase and restriction enzymes were obtained from New England Biolabs (USA). Primers were synthesized by Shanghai Sangon Biological Engineering Technology & Services. Their sequences are provided in Supplementary Tab. 1. DEAE Sepharose Fast Flow and Sephacryl S-200 HR were purchased from GE Healthcare life sciences (USA). Protein marker was purchased from Solarbio (Beijing, China).

FastAP^TM^ Thermosensitive Alkaline Phosphatase (FastAP) was purchased from Fermentas (EF0651) and was dissolved in a solution of 20 mM HEPES-NaOH (pH 7.4), 1 mM MgCl_2_, 0.1 mM ZnCl_2_, and 0.1% (v/v) Triton X-100. FastAP reaction buffer contained 100 mM Tris-HCl (pH 8.0, 37 °C), 50 mM MgCl_2_, 1 M KCl, 0.2% (v/v) Triton X-100 and 1 mg ml^−1^ BSA. T4 Polynucleotide Kinase (T4 PNK) was purchased from Fermentas (EK0037) and was dissolved in a solution of 20 mM Tris-HCl (pH 7.5), 25 mM KCl, 0.1 mM EDTA, 2 mM DTT and 20% glycerol to a concentration of 10 U μl^−1^. The reaction buffer included 500 mM Tris-HCl (pH 7.6, 25 °C), 180 mM MgCl_2_, 50 mM DTT and 1 mM spermidine.

T4 DNA Ligase was purchased from Fermentas (EL0014) and was stored in stock buffer composing of 20 mM Tris-HCl (pH 7.5), 1 mM DTT, 50 mM KCl, 0.1 mM EDTA and 50% (v/v) glycerol. Reaction buffer was including 400 mM Tris-HCl (pH 7.5), 100 mM MgCl_2_, 100 mM DTT and 5 mM ATP (pH 7.8, 25 ^o^C).

All stock solutions were stored at −20 °C, and all the stock nucleic acids (NAs) solutions were stored at −80 °C before use.

### Expression, purification and activation of recombinant pepsin (rP) and mutant pepsin (mP)

According to the sequence of porcine pepsinogen cDNA (http://www.ncbi.nlm.nih.gov/), two primers (PF and PR, detailed sequences are given in Supplementary Tab. 1) were designed to amplify the ORF (Open Reading Frame) from the pepsin cDNA. The ORF was amplified by PCR and cloned into the *Eco*RI and *Not*I sites of the vector **pPICZα A**. Then, the vector was transformed into competent *E. coli* TOP10 cells and sequenced by BGI (Beijing Genomics Institute, China). The recombinant plasmid **pPICZα A** was named **pPPGA**. **pPPGA** was linearized with *Pme*I and electroporated into competent *Pichia Pastoris* X-33 cells according to the manufacturer’s instructions (Invitrogen). Transformants were screened on YPDS (Yeast Extract Peptone Dextrose Medium with Sorbitol) plates with 100 μg ml^−1^ Zeocin. High expression strains were screened by Zeocin gradient according to the manufacturer’s instructions (Invitrogen). Genomic DNA from recombinant strains was extracted, and the presence of the pepsinogen gene was confirmed by PCR using the universal primers 5′ AOX1 and 3′ AOX1.

For extracellular expression of pepsinogen, a recombinant clone of *Pichia Pastoris* was pre-cultured in YPD medium and then proliferated in buffered glycerol-complex media (BMGY) at 30 °C in an incubator shaker at 220 r.p.m. After 20 h, the medium was replaced with BMMY (buffered methanol-complex medium) and incubated for another 96 h. 0.5% methanol was added every 24 h to maintain induction conditions. Cultures were centrifuged at 5,000 r.p.m for 5 min at 4 °C, and the supernatant was collected.

Pepsinogen was precipitated by slow addition of ammonium sulphate to 60% saturation. After dialysis against 25 mM of phosphate buffer (pH 7.0; buffer I) overnight at 4 °C, the protein was applied to a DEAE Sepharose FF column (GE Healthcare Life Sciences, 5 × 10 cm, flow rate 5 ml min^−1^). The column was gradient eluted by buffer I containing 0 to 0.5 M of NaCl. The active fraction in 0.5 M of NaCl was dialyzed against 25 mM of phosphate buffer (pH 7.0) containing 0.15 M NaCl (buffer II), then was applied to Sephacryl S-200 HR (1.6 × 60 cm) and eluted by buffer II at a flow rate of 0.5 ml min^−1^. The active fraction was collected, desalted and lyophilized as purified recombinant pepsinogen (rPG). Fraction collections during column chromatography were monitored by a pepsin activity assay^12^. All procedures were performed at low temperatures (0–4 °C).

To activate rPG, rPG was diluted in 0.01 M of HCl (pH was approximately 2.0) and incubated at 25 °C for 0.5 h. The mixture was neutralized by the addition of 1 M sodium acetate (pH 5.3). The prosegment and salt were removed by ultrafiltration using a YM-10 membrane (Millipore, USA).

Mutant pepsinogen harboured two amino acids changes at sites 32 and 215 of pepsin and was produced by site-directed mutagenesis^11^,^24^. The Asp at site 32 was changed to Ala using primers 32F1, 32R1, 32F2 and 32R2 (see sequence details in Supplementary Tab. 1), while Asp at site 215 was changed to Ala using primers 215F1, 215R1, 215F2 and 215R2 (see details in Supplementary Tab. 1). The subsequent cloning, purification and activation procedures were the same as for native rPG.

Protein concentration was determined by Bradford assay (1976)^25^ using bovine serum albumin as a standard. During enzyme purification, elution peaks were measured by absorbance at 280 nm.

The molecular weight of recombinant and mutant pepsins were analysed as described by Laemmli (1970)^26^ using a 5% (w/v) stacking gel and a 12% (w/v) separating gel. The molecular masses of the proteins were estimated by calibration of the gels with a protein marker.

The purities of recombinant and mutant pepsin were analysed by HPLC. The HPLC conditions were described as follows: TSK gel G2000SWxl UV 280 nm; flow rate, 0.5 ml min^−1^; mobile phase, 25 mM Na_2_HPO_4_-NaH_2_PO_4_ buffer (pH 6.0, containing 25 mM NaCl).

### Digestion of nucleic acids by gastric juice

Gastric juices of different pHs were adjusted by the addition of 25 mM NaH_2_PO_4_ buffer solution (pH 8.0) to 3.8. Then, original or pH-adjusted gastric juices were added to NA solutions at a ratio of 3:1 (v/v). Digestions of NAs by gastric juice were carried out at 37 °C for 3 h. After digestion, the pH was adjusted to 7.0–8.0 in preparation for electrophoresis.

### Digestion of nucleic acids by pepsin

Stock solutions of pepsin, NA, digestion buffer and H_2_O were mixed together at a ratio of 2:3:5:10 (v/v/v/v). The mixture was reacted at 37 °C for 0–24 h. Different concentrations of pepsin were diluted pepsin stock solution by H_2_O before the reaction. Different concentrations of NaCl in buffer solutions were prepared in storage buffer with an appropriate quantity of NaCl. After the reaction, NAs were immediately extracted using the phenol-chloroform method^27^. The upper fraction of the solution containing NAs was kept for electrophoresis. The ssDNA of 1 μM such as S82 , S70 and 59 nt long sequences were also digested as above.

### Digestion of nucleic acids by recombinant pepsin (rP) and mutant pepsin (mP)

rP and mP were dissolved in H_2_O at a concentration of 0.25 mg ml^−1^. Storage solutions of rP NA and digestion buffer were mixed together at a ratio of 12:3:5 (v/v/v). The mixture was reacted at 37 °C for 12 h. mP was reacted with NAs in the same manner as rP. After the reaction, NAs were extracted and electrophoresed as described above.

### Inhibition of pepsin by an alkaline solution

Four microliters of pepsin storage solution (40 mg ml^−1^) was adjusted to pH 8.0 using 5 mM NaOH and maintained at room temperature for 0.5 h to inactivate pepsin. Then, 10 μl of storage buffer (pH 2.0), 6 μl of λ DNA and 20 μl of H_2_O were added. The final pH of the mixture was 3.8, as measured by a S20K pH meter (Mettler-Toledo, Shanghai, China). For the control group, H_2_O was added instead of NaOH. The mixture was then reacted at 37 °C for 2 h and analysed by electrophoresis as described above.

To inhibit pepsin, 30 μl of gastric juice was added to pepsin solution and the pH was adjusted to 8.0 by the addition of 5 mM NaOH. After 0.5 h, inactivated gastric juice was readjusted to pH 3.8 with stored buffer (pH 2.0), then 30 μl of the gastric juice (pH 3.8) was mixed with 10 μl of NA storage solution and reacted at 37 °C for 3 h. Extraction and analysis of NAs was carried out as described above.

### Phosphorylation and dephosphorylation

S59CGTT (3′ FITC) was digested by 8 mg ml^−1^ pepsin at pH 3.8 NaH_2_PO_4_ buffer (25 mM NaH_2_PO_4_ and 200 mM NaCl) for 12 h then extracted using the phenol-chloroform method. Five microliters of S59CGTT (3′ FITC) products (2 μM), 1 μl of 10 × T4 PNK buffer, 1.25 μl of ATP (10 mM) and 0.75 μl of 10 U μl^−1^ T4 Polynucleotide Kinase (T4 PNK, Fermentas) were mixed together. Finally, 2 μl of H_2_O was added. Reactions were carried out at 37 °C for 0.5 h, then were heated to 75 °C to inactivate the enzyme.

Dephosphorylation reaction was similar to phosphorylation reaction, except S59CGTT (without FITC labeled 3′end) as the substrate. T4 PNK was replaced with FastAP (FastAP^TM^ Thermosensitive Alkaline Phosphatase, Fermentas).

### Ligation

After phosphorylation, 2 μl of Sp20 fragment (10 μM), 2 μl of Syn-31 ssDNA (10 μM), 1 μl of T4 DNA ligase buffer and 2.5 μl of H_2_O were added to 10 μl of phosphorylated solution. The mixture was kept at 90 °C for 5 min, then cooled to room temperature. Next, 2 μl of PEG 4000 and 0.5 μl of T4 DNA ligase were added to a final volume of 20 μl. The molar ratio of Syn-31: Sp20: S59CGTT (3′ FITC) was 2 : 2 : 1. The ligation was kept at room temperature for 1 h then heated to 65 °C for 15 min to inactivate T4 DNA ligase enzyme. A 20% denaturing acrylamide gel was used for electrophoresis.

For the dephosphorylated products, Sp20 and Syn-31 were replaced by Sp14 and Syn-16, and other ligation conditions were the same as for phosphorylation.

### Inhibition of pepsin by pepstatin A

To inhibit pepsin, 1 μl of pepstatin A (10 mg ml^−1^) was added to 10 μl of pepsin (0.4 mg ml^−1^) and incubated at 37 °C for 0.5 h. Pepsin, pepstatin A, NAs, digestion buffer and H_2_O were mixed together at a ratio of 10:1:6:10:13 (v/v/v/v/v), then reacted at 37 °C for 5 h. After digestion, NAs were extracted and analysed as described above. The pepsin-negative group contained DMSO instead of pepstatin A solution. To inhibit pepsin in gastric juice, original gastric juice was diluted 1,000 times by 100 mM NaH_2_PO_4_ (pH 3.0, containing 800 mM NaCl), then 10 μl of pepstatin A (10 mg ml^−1^) was added to 10 μl of gastric juice and incubated at 37 °C for 0.5 h. Diluted gastric juice, pepstatin A, NAs and H_2_O (1:1:1:1, v/v/v/v) were mixed and reacted for 1 h. Electrophoresis conditions were the same as for active pepsin.

### Hydrolysis of nucleic acids by Cathepsin D or trypsin

Digestion of λ DNA by Cathepsin D or trypsin was similar to the protocol used for pepsin. λ DNA at a concentration of 45 μg ml^−1^ was treated by 9 mg ml^−1^ Cathepsin D in a 25 mM NaH_2_PO_4_ buffer solution (pH 3.5) at 37 °C for 24 h.

For trypsin, 45 μg ml^−1^ of λ DNA was treated by 1 mg ml^−1^ of trypsin in a 25 mM Tris-HCl buffer solution (pH 8.5) or 25 mM NaH_2_PO_4_ buffer solution (pH 3.5) at 37 °C for 5 h.

### Kinetic measurements

To measure *K*_m_ and *k*_cat_ values, a synthetic substrate of 82 nt labelled by FITC at its 5′ end was employed. Final concentration of S82 was 1 μM. Pepsin in concentration range of 2.85 μM–115 μM were incubated at 37 °C (pH 3.0) for various times to determine the initial rates. After degradation, samples were extracted by phenol and chloroform as described above, and 10 μl supernatant was electrophoresed on a 20% denaturing polyacrylamide gel. The images were captured by Image Lab 3.0, and a relative amount of DNA in different bands was analyzed using software of Molecular imager Gel Doc XR+ imaging system. Then, we used Lineweaver-Burk to calculate the *K*_m_ and derived the *k*_cat_ value. The results are reported as the mean of three independent experiments for each concentration of S82.

### Agarose gel electrophoresis

Extracted NAs were electrophoresed as described previously^28^. Concentrations of NAs were measured by NanoDrop ND 2000 (Thermo Fisher Scientific). NA solution (8.0 μl) was electrophoresed on a 0.8% agarose gel by DYCP-31DN (Liuyi, Beijing, China) under 100 v using DYY-6C (Liuyi, Beijing, China) as power supply. Gel was stained by 0.005‰ EB (Ethidium bromide) for 20 min, and then imaged by GEL DOC XR + (Bio-Rad, CA, US). Data were analyzed by Image Lab Software Version3.0 (Bio-Rad, CA, US).

### Polyacrylamide gel electrophoresis

Extracted NAs were electrophoresed on native or denaturing polyacrylamide gel as described previously^29^. NA was performed on a 20% polyacrylamide gel by DYCZ-24F (Liuyi, Beijing, China) under 350 v. Other conditions were the same as agarose gel electrophoresis as described above.

## Additional Information

**How to cite this article**: Liu, Y. *et al.* Digestion of Nucleic Acids Starts in the Stomach. *Sci. Rep.* **5**, 11936; doi: 10.1038/srep11936 (2015).

## Supplementary Material

Supplementary Information
