# Supplementary material for: Digestion of Nucleic Acids Starts in the Stomach
Source: Sci Rep. 2015 Jul 14;5:11936. doi: 10.1038/srep11936 (PMC4500949; doi:10.1038/srep11936)
Supplement: Supplementary Information [file srep11936-s1.doc]

**Digestion of Nucleic Acids Starts in Stomach**

Yu Liu^1^, †, Yanfang Zhang^1^, †, Ping Dong^1^, *, Ran An^1^, Changhu Xue^1^, Yinlin Ge^2^, Liangzhou Wei^2^ & Xingguo Liang[[1]](#footnote-2), *

**Supplementary Information**

**Supplementary Figures**

**Supplementary Figure 1: Effect of pH on digestion of λ DNA by pepsin. a**, Pepsin (4.0 mg ml^-1^) was present. **b**, Pepsin was absent. Significant digestion of λ DNA was found when pH < 5.3 (Lanes 1-8, **a**). Compared with digestion by pepsin (**a**), the low pH (acidic condition) itself had almost no effect on digestion (**b**). Although breakage of λ DNA was found after 24 h in the absence of pepsin at pH 2.2 (Lane 2, **b**), the effect of pepsin on digestion was significant, especially within 2 h (comparison of Lane 1 in **a** with Lane 1 in **b**). The bands at pH 2.0 was light because the depurination was carried out at such a low pH and the depurination products were hard to be stained.

**Supplementary Figure 2: Effect of pH on digestion of salmon sperm DNA by pepsin. a**, Pepsin (4.0 mg ml^-1^) was present. **b**, Pepsin was absent. The reaction time was 2.0 h. For salmon sperm DNA, similar digestion results were obtained as those of λ DNA. Phosphate buffered saline with pH higher than 2.2 did not show any effect on the digestion of salmon sperm DNA (Lanes 2-7, **b**). However, when 4.0 mg ml^-1^ pepsin was added, significant digestion of NA was observed after 2 h (Lanes 2-7, **a**).

**Supplementary Figure 3: Inhibition effect of alkaline treatment of pepsin (a) and gastric juice (b) on salmon sperm DNA digestion.** 0.8% agarose gel was used for electrophoresis. In (**a**), the alkaline pretreated or non-pretreated pepsin was mixed with buffer (25 mM NaH_2_PO_4_, 200 mM NaCl) and the final pH was 3.8, and reaction time was 2 h. In (**b**), digestion was carried out at pH 3.8 for 3 h. Obviously, the digestion activity was almost lost after alkaline pretreatment for both pepsin (**a**) and gastric juice **(b)**.

**Supplementary Figure 4: Effect of pH on digestion of λ DNA by DNase I.** Digestion was performed with 1 U DNase I for 0.5 h, other conditions were the same as those in Fig. 2**a**. The optimum pH of DNase I on λ DNA was 7.0. DNase I was not inactivated at pH 8.0.

**Supplementary Figure 5: Effect of pH on the digestion of λ DNA by DNase II.** Digestion was performed with 0.04 mg ml^-1^ of DNase II, and other conditions were the same as those in Fig. 2**a**. DNase II showed activity even when pH was 7.0 (Lane 4). This was different from the case of pepsin, in which pepsin lost its activity when pH was higher than 6.0.

**Supplementary Figure 6: Determination of the purity of commercially provided, recombinant and mutant pepsin. a**, HPLC analysis of commercially provided pepsin. **b**, SDS-PAGE analysis of commercially provided pepsin. **c**, HPLC analysis of recombinant pepsin. **d**, SDS-PAGE analysis of recombinant and mutant pepsin. **e**, HPLC analysis of mutant pepsin. HPLC conditions: TSKgel G2000SWxl, UV 280 nm; flow rate, 0.5 ml min^-1^; mobile phase, 25 mM Na_2_HPO_4_-NaH_2_PO_4_ buffer (pH 6.0, containing 25 mM NaCl). Purity of commercially provided pepsin was determined as 98.6% (**a**). The purity for both recombinant pepsin and mutant pepsin was determined to be higher than 98%. The SDS-PAGE analysis showed that the molecular weight of mutant pepsin was 37.6 KDa, which was consistent with the calculated one (**d**).

**Supplementary Figure 7: Time course of λ DNA or salmon sperm DNA digestion by pepsin. a**, λ DNA. **b**, Salmon sperm DNA. The reaction time was 0.5 h, 1 h, 1.5 h, 2 h, 5 h, 12 h and 24 h. Other conditions: 4.0 mg ml^-1^pepsin, NaH_2_PO_4_ buffer (pH 3.8, 200 mM NaCl), 37 °C. The digestion products of λ DNA (**a**) were all oligonucleotides with the length from several dozens to several hundred of nucleotides, while mononucleotide was not found even extending the reaction time to 24 h. For salmon sperm DNA (**b**), the results were similar to those of λ DNA, digestion products were oligonucleotides rather than mononucleotide.

**Supplementary Figure 8: Effect of NaCl on the digestion of plasmid pET-28a.** With the increase of NaCl, digestion of plasmid pET-28a slowed down, especially when the concentration of NaCl was higher than 320 mM (Lane 9). Moreover, almost no digestion occurred when NaCl was higher than 400 mM (Lane 11).

**Supplementary Figure 9: Effect of pepsin concentration on digestion of λ DNA**. Pepsin concentrations were from 1.0 mg ml^-1^ to 0.1 μg ml^-1^. Digestion rate of λ DNA depended greatly on the concentration of pepsin. For 0.1 mg ml^-1^ of pepsin, the digestion slowed down, and only tiny digestion was observed within 2 h. However, the final product still showed an average length of about 300 bp after 24 h of digestion (Lane 4). The digestion was even observed after 24 h when pepsin concentration was as low as 1.0 μg ml^-1^ (Lane 8). When the concentration of pepsin decreased to 0.1 μg ml^-1^, no digestion was observed. The pH of digestion solution was 3.8.

**Supplementary Figure 10: Effect of hemoglobin on digestion of λ DNA**. Lane L, DNA ladder; Lane O, original λ DNA; Lane C, λ DNA and Hb (100:1, M/M), a control in the absence of pepsin; Lane 1, λ DNA hydrolyzed by pepsin without Hb; Lanes 2 to 5, λ DNA and Hb (Hb : λ DNA = 100:1, 1,000:1, 10,000:1 and 16,000:1; M/M) hydrolyzed by pepsin. Comparing Lane 1 (without Hb) with Lanes 2-5 (with Hb), we concluded that NAs could be digested efficiently by pepsin in the presence of Hb, and protein did not show inhibition effect on NA digestion. The result strengthened our suggestion that NAs could or tended to be digested by pepsin even ingested together with protein.

**Supplementary Figure 11: Kinetic analysis of pepsin using S82 as the substrate**. **a,** Time course of a reaction in the case of 115 μM pepsin. **b,** Determination of *K*_m_ and V_max_ for S82 using the Line weaver-Burk plot, the kinetic parameters were calculated as: V_max_ = 0.035 μM^-1^ min^-1^, *K*_m_ = 55.6 μM, *k*_cat_/*K*_m_ =1.05×10^-2^ s^-1^ mM^-1^. The data for drawing weaver-Burk plot (**b)** were listed in **c**.

**Supplementary Table 1: Primers for cloning and mutating porcine pepsinogen**

**Supplementary Material and Methods**

**Hydrolysis of** λ **DNA by DNase I or DNase II.** DNase I was purchased from Fermentas (EN0521) and was stored in buffer solution (pH 7.5, 50 mM Tris-acetate, 1 mM CaCl_2_ and 50% glycerol). DNase II was purchased from Sigma-Aldrich (D4138) and was dissolved in water to 4 mg ml^-1^. Enzyme/NAs/digestion buffer/H_2_O were mixed at the ratio of 2:3:5:10 (v/v/v/v) for DNase II or 1:3:5:11 (v/v/v/v) for DNase I. Other digestion and analysis conditions were the same as those for pepsin digestion.

**Hydrolysis of nucleic acids together with Hb.** Hemoglobin (Hb) stock solution was prepared to contain 40 mg ml^-1^ Hb (Sigma-Aldrich Co., Ltd.) in 65 mM HCl. Hb stock solution was diluted by 65 mM HCl to 155 μM, and then diluted to 95.1 μM, 9.51 μM and 0.951 μM by water. Then they were mixed with the same volume of λ DNA (300 μg ml^-1^, 9.51 nM) to produce a series of mixture Hb and λ DNA. Ratios of Hb/λ DNA were 10,000:1, 1,000:1 and 100:1 (M/M). To get higher concentration of Hb (Hb/λ DNA=16,000:1), we mixed the same volume of Hb solution (155 μM) and λ DNA (9.51 nM). Then 10 μl of digestion buffer (pH 3.0, 200 mM NaCl), 6 μl of H_2_O and 4 μl of pepsin stock solution were added. Digestion and electrophoresis were then carried out as described in “methods” section.

1. College of food science and engineering, Ocean University of China, Qingdao 266003, China. ^2^ Department of Biochemistry and Molecular Biology, Qingdao University Medical College, Qingdao 266003, China. † These authors contributed equally to this work. * Present address: College of food science and engineering, Ocean University of China, Qingdao, 266003, P.R.China. Correspondence and requests for materials should be addressed to P.D. (dongping@ouc.edu.cn) or X.L. ([liangxg@ouc.edu.cn](mailto:liangxg@ouc.edu.cn)). [↑](#footnote-ref-2)
